# Steam Reforming of Ethanol to Acetaldehyde and Acetone Over Al‐Doped ZnO Catalysts

**DOI:** 10.1002/cssc.202501925

**Published:** 2026-01-05

**Authors:** Astrid Sophie Müller, Lars Malte Alfes, Michael Fechtelkord, Martin Muhler

**Affiliations:** ^1^ Laboratory of Industrial Chemistry Department of Chemistry and Biochemistry Ruhr University Bochum Bochum Germany; ^2^ Institute of Geosciences Ruhr University Bochum Bochum Germany

**Keywords:** Al doping, decarboxylative ketonization, heterogeneous catalysis, steam reforming of ethanol, zinc oxide

## Abstract

Ethanol steam reforming with a water to ethanol molar ratio of 7:1 was investigated over pure ZnO and Al‐doped ZnO catalysts with up to 10 mol% Al^3+^ synthesized via coprecipitation. This synthesis route yielded wurtzite ZnO, with Al being incorporated into the ZnO lattice at low doping levels. Al doping was found to alter the reaction pathway of ethanol steam reforming by suppressing the consecutive acetaldehyde conversion to acetic acid and further to acetone. Continuous kinetic experiments using a plug flow reactor resulted in almost full conversion and an acetone selectivity of 53% at 450°C over pure ZnO. Feeding acetaldehyde and acetic acid confirmed a consecutive multistep reaction network starting with ethanol dehydrogenation to acetaldehyde, followed by its conversion to acetic acid and a subsequent decarboxylative ketonization to acetone and CO_2_. Upon Al doping, the specific surface area increased by about a factor of two, but conversion was hardly changed. Instead, the acetaldehyde selectivity increased, whereas acetone and CO_2_ formation decreased, indicating that Al incorporation selectively suppresses ketonization. Overall, acetone formation via ethanol steam reforming was found to be a strongly structure‐sensitive reaction over Al‐doped ZnO, with its surface acid–base properties strongly depending on the Al content.

## Graphical abstract

The steam reforming of ethanol over ZnO was found to consist of the dehydrogenation to acetaldehyde followed by its conversion to acetic acid forming acetone, CO_2_ and H_2_ via decarboxylative ketonization. The selectivity to acetaldehyde of this structure‐sensitive consecutive reaction network was enhanced by Al doping of ZnO, which modified the acid–base properties, whereas pure ZnO favored acetone formation.

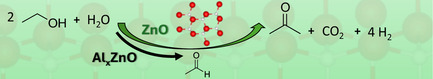

## 1 Introduction

Ethanol is an increasingly attractive renewable feedstock, which is primarily derived from sugarcane and corn, but also from economical low‐price sources such as cellulosic waste [1]. With its production steadily rising, bioethanol offers a sustainable alternative for various large‐scale applications, supporting the shift of chemical industry toward more environmentally friendly feedstocks. Bulk chemicals such as C_2_ – C_4_ olefins are highly relevant for the production of solvents, polymers, and drugs, and using bioethanol as feedstock can lead to the same variety of intermediates [2].

The ethanol steam reforming (ESR) to CO_2_ and H_2_ is a strongly endothermic process [3].

(1) $$C_{2}H_{5}\text{OH}+3H_{2}O\to \;2{\text{CO}}_{2}\;+\;6H_{2};\;\text{Δ}H_{298}^{0}=\;174\;{\text{kJ mol}}^{-1}$$

This process competes with undesired side reactions such as the endothermic formation of CO (Equation (2)) and the exothermic formation of CH_4_ (Equation (3)) as investigated by Aupretre et al. [4] Coking is also a commonly observed phenomenon during ESR. Ni‐based catalysts supported on Al_2_O_3_ or CeO_2_ show the highest activity in the ESR to H_2_ with Ni/CeO_2_ being the most promising candidate due to the suppression of carbonaceous residues [5, 6].

(2) $$C_{2}H_{5}\text{OH}\;+\;H_{2}O\to \;2\text{CO}+\;4H_{2};\;\text{Δ}H_{298}^{0}=\;256\;{\text{kJ mol}}^{-1}$$

(3) $${C_{2}H_{5}{\text{OH + 2H}}_{2}{\text{→ 2 CH}}_{4}{\text{+H}}_{2}{\text{O; ΔH}}_{\text{298}}^{\text{0}}\text{= − 157 kJ mol}}^{-1}$$

Depending on the reaction conditions, especially the water content in the feed [7] and the catalyst used, the product distribution can be controlled to achieve industrially relevant products such as unsaturated hydrocarbons (ethylene, propylene, and isobutene) and oxygenates such as 1‐butanol, acetaldehyde, and acetone [8, 9, 10]. For this purpose, many authors reported several pure or mixed metal oxides to achieve a high conversion of ethanol to valuable products [11, 12]. The synthesis of butadiene was extensively studied in the last century [13, 14]. In the Ostromislenski process, ethanol is converted to butadiene via acetaldehyde in a two‐step process, whereas the Lebedev process is a one‐step route employing a mixed catalyst. In both processes, crotonaldehyde represents the key intermediate [15, 16]. Different product distributions comprising mainly ethylene, acetone, acetaldehyde, H_2_ and CO_2_ with high ethanol conversion were found for pure [17] and supported CeO_2_ [18] as well as for doped catalyst systems such as Co/Ce_90_Ga_10_O_*x*_ [19]. Pure ZrO_2_ and mixed oxides, especially Zn_*x*_Zr_1‐*x*_O_2‐*y*_ [20] and ZnZr_*y*_O_z_ [21, 22], yielded acetone as main intermediate in the formation of isobutene. The underlying mechanism of these catalysts has been intensively studied by Bell and coworkers, where the ketonization of acetic acid is described as key step [22]. Several mechanistic studies were performed without directly identifying decarboxylative ketonization as main reaction pathway to acetone: pure ZnO showed an intrinsically high ethanol conversion of 57.9% at 400°C [12] that can be modified by adding other oxides to obtain ZnO‐CoO/Al_2_O_3_ [7], ZnO‐CaO [23, 24], or ZnFe mixed oxide catalysts [12]. Thus, ZnO‐based catalysts are characterized by high catalytic activity and selectivity and considered sustainable compared with Co, Ce, or other noble metal‐based catalysts.

In this work, Al‐doped ZnO was investigated in the ESR with an excess of water. Aldoped ZnO has been shown to be very promising for the synthesis of oxygenates [7, 12, 24]. This catalyst is well established concerning its synthesis and characterization [25, 26, 27]. After coprecipitation and washing, the monoclinic hydrozincite Zn_5_(OH)_6_(CO_3_)_2_ [28] was obtained, into which increasing amounts of Al up to 10 mol% were incorporated. Calcination at 320°C led to ZnO in the wurtzite structure. ZnO has been used for decades as an important component of the industrially used methanol synthesis catalyst and has been extensively studied with respect to its surface and bulk chemistry [29, 30]. Investigations concerning its microstructural properties were not only performed for pure ZnO but also for the mixed ZnO/Al_2_O_3_ [31]. ZnO is well‐suited for dehydrogenation reactions due to its basic character, and the substitution of Zn^2+^ cations by Al^3+^ cations as dopant impacts the surface acidity of ZnO, leading to a unique product distribution in the conversion of ethanol with excess water to acetaldehyde and acetone.

## 2 Results and Discussion

### 2.1 Characterization

**FIGURE 1:**
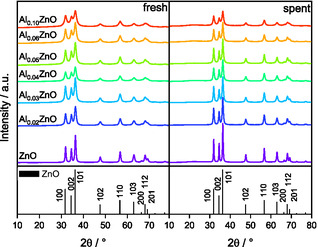
XRD patterns before (left) and after (right) ethanol steam reforming over the calcined catalysts.

The elemental composition of the dried precursors is summarized in Table S1. The X‐ray diffraction (XRD) analysis of the dried precursors shows that the observed reflections can primarily be assigned to hydrozincite (Figure S1) [25, 32]. Minor structural differences observed in the diffraction patterns are attributed to the incorporation of Al^3+^ into the hydrozincite lattice. With increasing Al content, the intensity and sharpness of the reflection at 2*θ* = 35.6° increases, and a reflection at 2*θ* = 19° begins to emerge at an Al loading of 5%, which is typically forbidden in this space group [25] but becomes allowed due to the structural modifications introduced by Al^3+^. This observation aligns with findings by Mockenhaupt et al. [25] The calcination of the precursor resulted in the formation of pure ZnO (Figure 1) that adopts the wurtzite structure with sharp reflections indicative of high crystallinity [26, 32]. With increasing Al incorporation, slight shifts in the reflections at 2*θ* = 34° and 2*θ* = 36° toward higher angles are observed, suggesting lattice distortions. In contrast, reflections around 2*θ* =  70° corresponding to the {112} and {201} lattice planes are weakened or absent. The ICP‐MS results of the calcined samples summarized in Table 1 validate the successful Al doping levels for all samples synthesized by coprecipitation. Additionally, XRD analysis of the spent catalysts after ESR at 400°C shows that the wurtzite phase remains stable with no signs of phase transformation or by‐phase formation. However, the reflections become sharper due to the increased crystallinity upon prolonged exposure to reaction conditions.

**TABLE 1:** ICP‐MS results for the calcined catalysts and crystallite sizes obtained by applying the Scherrer equation to the reflection at 2*θ* = 46° of the fresh and spent catalysts.

Catalyst	*x*(Zn) (%)	*x*(Al) (%)	*D* (Å); (fresh)	*D* (Å); (spent)
ZnO	100.0	0	115.4	165.7
Al_0.02_ZnO	98.0	2.0	82.4	127.2
Al_0.03_ZnO	97.2	2.8	73.4	103.3
Al_0.04_ZnO	95.7	4.3	70.6	103.6
Al_0.05_ZnO	95.0	5.0	73.7	113.4
Al_0.06_ZnO	94.0	6.0	57.1	103.8
Al_0.10_ZnO	89.8	10.2	56.1	103.6

The crystallite sizes of the fresh and spent catalysts were derived using the Scherrer equation based on the {102} reflection at 2*θ* = 47°. As summarized in Table 1, the crystallite sizes decrease with increasing Al content from 115.4 to 56.1 Å. After reaction, however, the crystallite sizes were larger by ≈40 Å for all samples. Notably, the decrease in the initial crystallite size is not uniform as a function of the Al content but can be grouped into two ranges of clusters: 3%–5% and 6%–10% Al, as the crystallite size remains nearly constant within the respective range.

^27^Al MAS‐NMR spectra of the samples exhibit similar features (Figure S2). The main resonance of the central transition in the spectra is centered at about 10 ppm, broad and with an asymmetric extension toward lower values resulting from distributions of quadrupolar parameters of ^27^Al, arising from the disordered nature of the AlO_*x*_ species. Typically, this resonance is caused by octahedrally coordinated Al (Al^VI^). A second signal arises at about 80 ppm, changing in relative intensity and linewidth. According to previous studies [33], this peak is assigned to tetrahedrally coordinated Al (Al^IV^). According to Stebbins et al. [33, 34], fivefold coordinated aluminum (Al^V^) produces a signal around 40–60 ppm in aluminosilicate glasses. The broad amorphous signal in this region can be assigned to this fivefold Al coordination. The relative intensity of Al^IV^ and the signal linewidth seems to correlate with the composition. Narrower lines and also a stronger amplitude appear at low Al contents and imply a more crystalline and ordered environment of Al^IV^. A similar linewidth narrowing depending on Al content also applies to Al^VI^, but here the linewidth decreases with increasing Al content, i.e., the Al^V^ resonances show no direct correlation to composition.

The BET and BJH analysis of the calcined samples indicate an increase in specific surface area with rising Al content, accompanied by a decrease in pore radii. These results are consistent with the crystallite size trends observed by XRD. The adsorption isotherms (Figure S3) correspond to Type IV in the IUPAC classification with hysteresis loops of Type H1 and H2, indicating the presence of mesopores [35] (Table 2).

**TABLE 2:** BET and BJH analysis of the N_2_ physisorption results for the calcined samples.

Catalyst	*S*_BET_ (m^2^ g^−1^)	*V*_p_ (cm^3^ g^−1^)	*r*_p_ (nm)
ZnO	35.3	0.14	7.0
Al_0.02_ZnO	60.7	0.17	4.6
Al_0.03_ZnO	55.6	0.15	8.0
Al_0.04_ZnO	54.8	0.14	3.7
Al_0.05_ZnO	83.7	0.22	4.0
Al_0.06_ZnO	61.8	0.17	8.2
Al_0.10_ZnO	88.1	0.22	1.9

DRIFTS measurements using pyridine as probe molecule were conducted to investigate acidity [36]. The spectra in Figure S4 show increased intensity for the bands at 1609, 1592, 1578, and 1442 cm^−1^ with increasing Al content, which correspond to Lewis acidic sites (LAS, 1609 cm^−1^, 1492 cm^−1^), Brønsted acidic sites (BAS, 1592 cm^−1^), and adsorbed pyridine (Pyr_ads_, 1578 cm^−1^, 1442 cm^−1^) on both types of sites [37, 38]. The observed pyridine bands can be correlated with the coordination of the exposed Al ions. The band at 1592 cm^−1^ is attributed to octahedrally coordinated Al (Al^VI^), and the band at 1609 cm^−1^ is assigned to pyridine bound to an Al^VI^‐Al^IV^ cation pair [39]. This effect is particularly pronounced in ZnO samples with higher Al doping levels (6%, 10%), indicating that Al doping significantly enhances the number of both Lewis and Brønsted acidic sites.

### 2.2 Catalytic Properties

**FIGURE 2:**
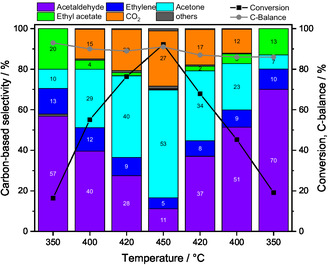
Conversion and carbon‐based selectivities and carbon‐balance by feeding EtOH:H_2_O:He =  1:7:92, Q =  100 mL min^−1^ as a function of temperature over ZnO at 350°C, 400°C, 420°C, and 450°C holding each temperature for 3 h. The black and gray lines are intended to guide the eye. Others include propylene, CH_4_, HOAc, and butenes.

Figure 2 shows conversion and the carbon‐based selectivities derived from the volume fractions (Figure S5) during the stepwise temperature variation over pure ZnO. As temperature increases, ethanol conversion rises from 19% to a maximum of 96%, with acetaldehyde, acetone, and CO_2_ as the main products above 350°C. Ethanol dehydrogenation to acetaldehyde prevailed at lower temperatures, while acetone formation coupled with CO_2_ formation dominated at higher temperatures. Additionally, by‐products such as ethylene, ethyl acetate, CH_4_, propylene, and traces of butenes were observed above 420°C. This trend remains for the cooling branch, although conversion does not reach the same levels as during heating which implies that the ZnO catalyst is not stable above 420°C. This is also a result of the low calcination temperature of 320°C compared with the reaction temperatures above 350°C. The carbon balance deficit is attributed to unquantified long‐chain products in GC analysis and to carbon deposition. Carbonaceous deposits were identified by the color change of the catalyst from bright yellow (fresh) to gray (spent).

The product distribution suggests a complex reaction network over ZnO, in which various side reactions and consecutive reactions proceed leading to different oxygenates and olefins. The endothermic formation of acetone ($\text{Δ}H_{298\;K}^{0}$ = 99.2 kJ mol^−1^) is coupled with CO_2_ formation according to the reaction equation [12]
(4)

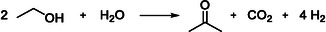

(5) $$\mathrm{CO}+H_{2}O⇋{\mathrm{CO}}_{2}+H_{2}$$

(6) $$C+H_{2}O\to \mathrm{CO}+H_{2}$$

With increasing temperature, the selectivity to acetaldehyde decreases from 57% to 11%, while the acetone selectivity reaches 53% at 450°C. The selectivity to CO_2_ exceeds the stochiometric ratio to acetone for each temperature. Therefore, acetone formation is not the only source for CO_2_, and processes such as the water gas shift reaction (WGSR) (Equation (5)) and coke gasification (Equation (6)) must be considered to take place. The decline in ethyl acetate selectivity from 20% to 0% can be attributed to thermodynamics, since its formation from ethanol and acetic acid is exothermic (Equation (7)) [10].
(7)

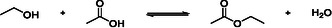




Unsaturated hydrocarbons such as ethylene, propylene, and butene were also observed. Although dehydration to ethylene is an endothermic reaction, its selectivity decreased at elevated temperatures from 13% at 350°C to 5% at 450°C, likely due to blocking of active sites that impairs the dehydration pathway. Propylene is formed above 400°C reaching a carbon‐based selectivity of 3% at 450°C, while butene is observed only in traces with a selectivity of 0.1% at 420°C and 0.4% at 450°C. Both olefins may be considered as consecutive products formed from acetone [22]. CH_4_ originates from acetic acid decomposition (Equation (8)), with CH_4_ selectivity increasing from 0% to 1.2% with increasing temperature.



(8)

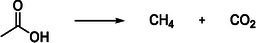




**FIGURE 3:**
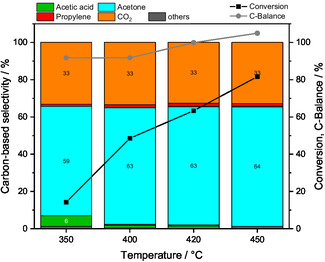
Conversion and carbon‐based selectivities and carbon‐balance by feeding acetaldehyde:H_2_O:He =  1:7:92, Q =  100 mL min^−1^ over ZnO at 350°C, 400°C, 420°C, and 450°C holding each temperature for 3 h. The black and gray lines are intended to guide the eye. Others include ethylene, CH_4_, and butenes.

Acetaldehyde is assumed to be the key intermediate when converting ethanol to acetone. Therefore, a feed consisting of 1% acetaldehyde and 7% water was used instead of 1% ethanol and 7% water. The results of feeding an acetaldehyde‐H_2_O mixture over ZnO (Figure 3) show an acetaldehyde conversion increasing from 17% to 82% with increasing temperature and acetone as the main product with an acetone selectivity around 60% for each temperature. The observed by‐products were acetic acid, propylene, methane and traces of butene. While the carbon‐based selectivities of acetone, CO_2_, propylene, and methane increased with increasing temperature, the selectivity of acetic acid decreased.

These results indicate a crucial role of acetaldehyde in the consecutive reaction network, since it serves as an intermediate in the formation of acetic acid and acetone (Equation (9)). This consecutive reaction network was previously proposed previously by Bell and coworkers and is found in detail elsewhere [22].



(9)

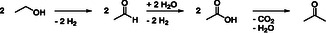




**FIGURE 4:**
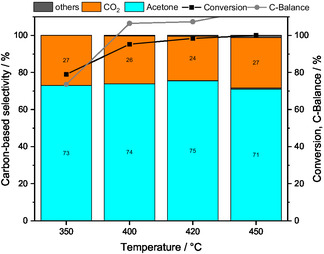
Conversion and carbon‐based selectivities and carbon‐balance by feeding HOAc:H_2_O:He =  1:7:92, Q =  100 mL min^−1^ over ZnO at 350°C, 400°C, 420°C, and 450°C holding each temperature for 3 h. The black and gray lines are intended to guide the eye. Others include CH_4_ and butenes.

The role of acetic acid as second key intermediate was investigated by feeding a mixture consisting of 1% acetic acid and 7% water. The C‐balance deviations are due to larger error bars of acetic acid in GC analysis. The results of the step experiment over ZnO (Figure 4) show an increasing conversion of acetic acid from 79% at 350°C to 100% at 450°C with acetone and CO_2_ as the main products. The ratio of the volume fractions of acetone to CO_2_ is near one at each temperature. Above 400°C, by‐products such as butenes and CH_4_ are observed with increasing selectivities with the increasing temperature. These results suggest that the final step in Equation (9), the decarboxylative ketonization, is another key step in acetone formation. Based on the higher conversion of acetic acid compared with acetaldehyde observed in the corresponding feeding experiments (Figures 3 and 4), it can be concluded that the oxidation of acetaldehyde to acetic acid is slower than the decarboxylative ketonization of acetic acid to acetone. The identification of the consecutive reaction network (Equation (9)) is supported by the results of residence time variation experiments using different masses of ZnO (Figure S6): when feeding an ethanol–water mixture, longer residence times favor acetone formation.

**FIGURE 5:**
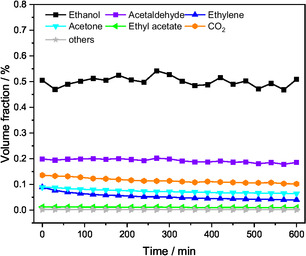
Effluent volume fractions during ethanol steam reforming using EtOH:H_2_O:He =  1:7:92, Q =  100 mL min^−1^ over pure ZnO at 400°C for 10 h. Others include propylene, CH_4_, HOAc, and butenes.

The long‐term stability at 400°C was evaluated over a period of 10 h and is shown exemplarily for pure ZnO in Figure 5. All volume fractions were essentially constant expect ethylene, which decreased from 0.08% to 0.04%.

**FIGURE 6:**
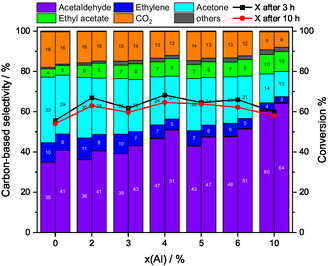
Conversion and selectivities of the Al_x_ZnO catalysts by feeding EtOH:H_2_O:He =  1:7:92, Q =  100 mL min^−1^ for 10 h at 400°C. The left column refers to the carbon‐based selectivity after 3 h and the right column after 10 h TOS. Ethanol conversion is shown as black squares (after 3 h) and red dots (after 10 h). Others include propylene, CH_4_, HOAc, and butenes.

The carbon‐based selectivities after 3 h (left column) and 10 h (right column) are represented together with the corresponding conversion in Figure 6. Al doping of ZnO changes the product distribution compared with pure ZnO, while conversion did not show a notable trend and remained around 60% at 400°C for each level of Al doping. Thus, conversion is not correlated with the specific surface area, which is about a factor of 2.5 higher for the 10% Al‐doped catalyst. For all catalysts, a decrease in conversion over time is observed, with the Al_0.02_ZnO and Al_0.04_ZnO catalysts showing the largest activity loss of 5%, while pure ZnO and Al_0.10_ZnO remained almost constant, losing 1%. Comparing the selectivities after the first 3 h with those after 10 h TOS, a similar behavior across all levels of Al doping is observed: the formation of acetaldehyde and ethyl acetate increased, while the selectivity to acetone, CO_2_, and ethylene decreased.

**FIGURE 7:**
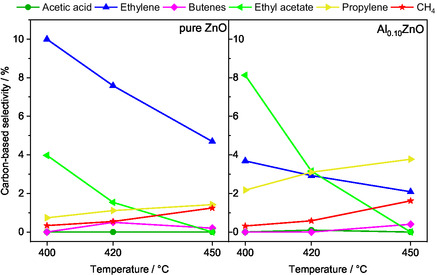
Carbon‐based selectivities of the by‐products formed over pure ZnO and Al_0.10_ZnO at 400°C, 420°C, and 450°C by feeding EtOH:H_2_O:He =  1:7:92, Q =  100 mL min^−1^.

Adding Al to ZnO induces two primary effects on the carbon‐based selectivity of the products. First, focusing on the first 3 h TOS, the product distribution shifts in favor of acetaldehyde when the Al content increases. Second, Al doping maintains the trend found for ZnO of primary product formation, but notably alters the by‐product distribution as shown in Figure 7 for ZnO and Al_0.10_ZnO. The conversion and selectivities of the temperature variation experiments over the Al‐doped ZnO catalysts are shown in Figure S7. With increasing Al content, the tendency for acetaldehyde to undergo consecutive reactions forming acetone and CO_2_ decreases. The acetaldehyde selectivity at 400°C after 3 h TOS increases from 35% for ZnO to 60% for Al_0.10_ZnO, while the acetone selectivity drastically decreases from 33% to 13%, respectively. Instead, a higher Al content promotes the formation of ethyl acetate and propylene, while the ethylene selectivity is lowered. As observed for pure ZnO, increasing temperatures lower the selectivity to ethylene and ethyl acetate, while the selectivity of the other by‐products such as propylene, methane and butene increases. This observation leads to the conclusion that the rate of the consecutive reaction of acetaldehyde to acetic acid is lowered by adding Al.

**FIGURE 8:**
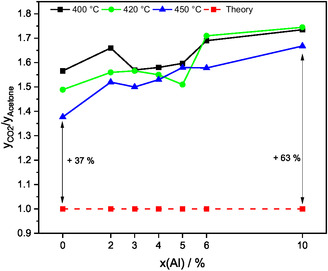
Ratio of the volume fractions of CO_2_ to acetone compared with the stoichiometrically expected ratio of 1:1 for various Al contents and temperatures.

Although CO_2_ formation is stoichiometrically coupled with acetone formation in a 1:1 ratio according to Equation (4), the kinetic measurements reveal that at 400°C, 56 to 73% more CO_2_ is detected (Figure 8). A higher reaction temperature decreases the excess of CO_2_, so that 37% to 63% more CO_2_ is detected at 450°C. The ratio of CO_2_ to acetone also depends on the Al doping level and overall increases by adding Al.

An offline GC analysis of the product gas stream at 420°C using ZnO was performed to quantify the amounts of H_2_ and CO_2_ by employing a gas mouse. The mole fractions of CO_2_ and H_2_ were found to be 0.183% and 0.707%, which corresponds to a ratio of 1:3.86 and a combined ratio of acetone:CO_2_:H_2_ of 1:1.56:6.04 as summarized in Table 3. Obviously, additional reaction pathways must be present which release CO_2_ and H_2_, presumably related to the decomposition of intermediates such as acetic acid and the steam reforming of carbonaceous deposits. The TG measurements of the spent catalysts in 20% O_2_/He shown in Figure S8 indicate that notable amounts of CO_2_ are released. Thermodynamic studies suggest that graphitic carbon species are deposited below 400°C when the steam to ethanol ratio is below 4.0 [40]. Similar to the findings of Alberton et al. [41], our reaction conditions differ from these thermodynamic predictions, suggesting that coke formation may be linked to kinetic limitations in the gasification process. The TG measurements of the spent catalysts show that Al‐doped ZnO up to a level of 6% exhibits the highest amounts of coke deposits, while pure ZnO and Al_0.10_ZnO do not show any additional CO_2_ release in O_2_ compared with inert conditions.

**TABLE 3:** Mole fractions of CO_2_ and H_2_ obtained by offline analysis and of CO_2_ and acetone obtained by online analysis at 420°C.

	y(CO_2_) (%)	y(H_2_) (%)	y(Acetone) (%)
Offline	0.183	0.707	—
Online	0.181	—	0.117

Finally, the question is addressed whether there is a correlation between the catalytic activities in ESR and the WGSR, which is assumed to occur via the formate mechanism over ZnO [42]. Table 4 summarizes the WGSR rates at 450°C and the acetone selectivities for selected catalysts, and Figure S9 shows the catalytic data. The results indicate that a higher catalytic activity in the WGSR correlates with an increased selectivity to acetone.

**TABLE 4:** WGSR reaction rate at 450°C compared with the acetone and acetaldehyde carbon‐based selectivities at 400°C, 420°C and 450°C.

Catalyst	***r***_**CO,450°C**_ **(mmol g**_**cat**_^**−1**^ **s** ^**−1**^)	Selectivity towards acetone (%)		
		400°C	420°C	450°C
ZnO	**57.3**	30.1	40.0	56.4
Al_0.05_ZnO	**17.3**	25.8	36.3	52.9
Al_0.10_ZnO	**8.91**	13.8	22.3	39.3

## 3 Discussion

Our results show that ethanol steam reforming over Al‐doped ZnO leads to a variety of products formed in parallel or consecutively. The dehydration of ethanol to ethylene occurs independently and is known to be catalyzed by strongly acidic sites such as those exposed by *γ*‐Al_2_O_3_ [43]. In addition to penta‐coordinated Al ions detected by ^27^Al NMR spectroscopy, *γ*‐Al_2_O_3_ contains highly coordinatively unsaturated tri‐ and tetra‐coordinated Al^3+^ cations located at corners and edges with strong Lewis acidic properties that are highly active in ethanol dehydration [44]. Despite the increased acidity with increasing Al content probed by pyridine adsorption, ethylene formation was found to decrease with increasing Al content. This observation suggests that Al doping primarily enhances weak and medium‐strength Lewis acidic sites, as confirmed by the pyridine DRIFTS measurements shown in Figure S4. ^27^Al NMR also confirms the presence of mainly highly coordinated Al, which results in an overall lower acidity. Additionally, ethylene formation decreases over time and at elevated temperatures for all catalysts, suggesting that active dehydration sites become rapidly blocked by coking under reaction conditions.

The kinetic results further indicate that two consecutive steps are involved in the formation of acetone: the dehydrogenation of ethanol to acetaldehyde and the subsequent conversion of acetaldehyde via acetic acid to acetone. The acetaldehyde feeding experiment confirms that ethanol dehydrogenation to acetaldehyde is the initial step for the formation of acetone, CO_2_, acetic acid, and propylene. The acetic acid feeding experiment further confirms the formation of acetone and CO_2_ from acetic acid, as described in the consecutive reaction network (Equation (9)). Acetone formation thereby serves as an indicator for the ability of acetaldehyde to undergo consecutive reactions. Al doping of ZnO was found to increase the selectivity to acetaldehyde and to lower the selectivity to acetone, raising the question of why the ability of acetaldehyde to react further is diminished.

**FIGURE 9:**
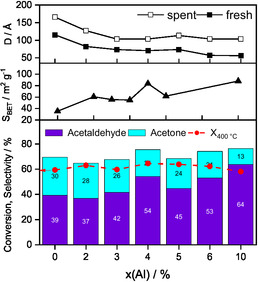
Specific surface areas and crystallite sizes of the catalysts compared with ethanol conversion and carbon‐based selectivities to acetone and acetaldehyde at 400°C.

To establish structure–activity correlations, the specific surface areas and crystallite sizes as determined by the Scherrer analysis are compared with ethanol conversion and the selectivities to acetone and acetaldehyde at 400°C for each dopant level of Al (Figure 9). Al doping increases the specific surface area significantly but does not affect ethanol conversion, indicating that ethanol conversion is not proportional to the exposed metal oxide surface. Obviously, the catalytic properties are neither significantly correlated with the specific surface area nor the crystallite size pointing to pronounced structure sensitivity.

The first step in ethanol steam reforming is the dehydrogenation reaction, which is well understood and aligns with several studies describing the dehydrogenation mechanism over ZnO, resulting in the formation of acetaldehyde and H_2_ (Equation (10)) [22, 23, 45].



(10)

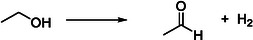




This reaction is catalyzed by a Zn‐O Lewis acid–base pair, which promotes the *α*‐H abstraction of ethanol. Acetaldehyde is identified as the most abundant reactive intermediate and is the first stage in the consecutive reaction to acetone. Acetaldehyde is known to adsorb on ZnO forming strongly bound acetate species that subsequently decompose or react further [46]. Several studies suggest that acetone formation from acetaldehyde involves surface‐bound acetate species [17, 19, 22]. According to the mechanism for acetone formation proposed by Bell and coworkers [22], acetaldehyde adsorbs on ZnO with its O‐atom binding to a Zn site and subsequently reacts with an OH group bound to Zn forming an acetal (Equation (11)). This OH group is either a surface‐terminating hydroxyl group or originates from H_2_O dissociation. This reaction results in the formation of an oxygen vacancy (V_O_) which is later refilled by H_2_O thereby restarting the catalytic cycle. Nakajima et al. [12] proposed a similar mechanism via acetate species where this nucleophilic attack is considered to be the rate‐determining step. At the same time, this step is strongly influenced by the Lewis acid–base properties of the metal oxide. The adsorbed acetal is then dehydrogenated to adsorbed acetate.



(11)

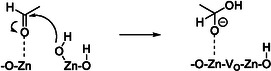




To investigate the tendency to form acetate species, the WGSR (Equation (5)) is a suitable reaction, since previous studies have shown that the WGSR over ZnO proceeds via the formation of bidentate formate species [42, 47, 48]. In the WGSR, formate decomposition is assumed to be the rate‐determining step [42, 47]. A correlation between the catalytic activity in the WGSR and the selectivity to acetone was indeed observed. These findings support the hypothesis that acetone is formed via acetate species following the adsorption of acetaldehyde on ZnO. Therefore, the doping of Al is assumed to impair the nucleophilic attack of the hydroxyl group resulting in a reduced formation of acetate species and thus acetone. This hypothesis will be addressed in future work using DRIFTS to study the adsorption of the probe molecules ethanol, acetaldehyde, and acetic acid and the subsequent conversion of the formed adsorbates as a function temperature. The previous discussion arrived at the conclusion that the Al doping of ZnO leads to a decrease in the basicity of the ZnO surface. These results are in agreement with the observations by Nakajima et al. for ZnO‐based catalysts compared with metal oxides with a less basic character [12]. Taking these results into consideration, the formation of acetate species from acetaldehyde is identified as the slower step in the consecutive reactions of acetaldehyde.

**SCHEME 1:**
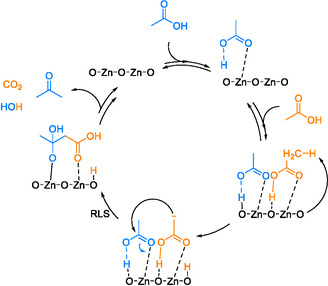
Decarboxylative ketonization over Zn_*x*_Zr_*y*_O_z_. Adapted from [22].

Acetate species formed from acetaldehyde are assumed to be hydrolyzed to acetic acid [46]. Acetic acid is observed as a minor side product (<1%, 350°C) when feeding ethanol but in larger amounts (6%, 350°C) when feeding acetaldehyde. At higher levels of Al doping, the acetone selectivity decreases, while the acetic acid selectivity becomes more pronounced. The acetic acid feeding experiment (Figure 4) shows that acetic acid is the second key intermediate in the consecutive pathway to acetone, with added Al inhibiting its further conversion compared with pure ZnO. Studies on the ketonization of acetic acid over different metal oxides show that acetone is the primary product coupled with CO_2_ and H_2_O via various acetate species [49, 50]. Overall, acetic acid reacts rapidly on ZnO and is only minimally observed when feeding ethanol. The adsorption of acetic acid results in molecular adsorption, hydroxy enolate, monodentate, or bidentate species depending on the metal oxide [50]. The decarboxylative ketonization of longchain carboxylic acids was investigated over different metal oxides such as MnO_2_, ZrO, and MgO and is claimed to occur via monodentate species on a single metal oxide site forming the corresponding ketone [51]. Similarly, Bell et al. describe the ketonization of acetic acid to form acetone which requires an abundance of basic sites and oxygen vacancies on Zr_*x*_Zn_*y*_O_z_ catalysts [22]. This proposed mechanism shown in Scheme 1 also involves monodentate acetate species from acetic acid bound to a single Zn center. In this process, acetic acid adsorbs via its carbonyl oxygen on a Zn cation, while the OH group is stabilized by an O anion of ZnO. One adsorbed acetic acid molecule can then react with a second adsorbed acetic acid molecule on a neighboring Zn site. Following a nucleophilic attack and several consecutive steps, acetone and CO_2_ are released.

All the studies on the ketonization of acetic acid indicate that relatively weak basic sites and the presence of monodentate acetate species are essential for high ketonization activity. Our findings that Al addition suppresses acetate species formation align with these mechanistic insights. Two significant effects of Al doping on the mechanistic pathway to acetone become evident. First, the decreased nucleophilicity of the hydroxyl groups results in higher selectivity to acetaldehyde, because it is less efficiently converted into acetic acid compared with pure ZnO. Second, the incorporation of Al^3+^ results in reduced basicity and increased Lewis acidity disfavoring the conversion of acetaldehyde to acetic acid. It is worth noting that Al^3+^ incorporation into the ZnO lattice is effective up to an Al mole fraction of 3% to 4% [25, 52]. Beyond these levels, Al^3+^ tends to segregate, forming Al‐rich phases such as the ZnAl_2_O_4_ spinel or even Al_2_O_3_. ^27^Al NMR data confirm the presence of both tetrahedrally and octahedrally coordinated Al with the fraction of octahedrally coordinated Al increasing at higher Al dopant levels, which may indicate the presence of X‐ray amorphous ZnAl_2_O_4_ or Al_2_O_3_. Both phases show a more acidic character, which is disadvantageous for the ketonization of acetic acid [49].

During the kinetic measurements, an excess of CO_2_ was observed for all catalysts, which increased with the Al doping level. GC measurements confirmed an acetone:CO_2_:H_2_ ratio of 1:1.56:6.04 for pure ZnO, which deviates significantly from the theoretical stoichiometric ratio of 1:1:4. This excess of CO_2_ is attributed to acetic acid decomposition and the gasification of coke deposits on the catalyst surface in the presence of the water vapor, which aligns with the observed gray to black color of the spent catalyst. Further studies are in progress using TG‐MS and TPO experiments combined with Raman spectroscopy to distinguish the two CO_2_ formation pathways. Our findings agree with several studies on methanol and ethanol steam reforming over Cu/ZnO/Al_2_O_3_ catalysts where coking is a commonly observed phenomenon [3, 53]. Overall, an increasing CO_2_ release under reaction conditions is observed with increasing Al doping level. We therefore suggest that coke gasification is enhanced by the addition of Al^3+^ [54], which promotes dissociative water adsorption and thus facilitates carbon gasification [55]. Besides the steam reforming of ethanol to CO_2_ and H_2_ also methane formation should be considered to explain the excess in CO_2_ [3] because of the observed increase in CH_4_ and CO_2_ selectivity at elevated temperatures (Figures 7 and 8, Tables S2–S4) pointing to the decomposition of acetic acid. The decomposition of acetate species on ZnO was described by Bowker et al. [46] in great detail.

These mechanistic insights provide the basis for further knowledge‐based improvement of the selectivity to acetone by tuning the surface acid–base properties via alkaline promoters and by using redox‐active cations favoring the formation of oxygen vacancies. Further kinetic and spectroscopic studies are in progress to prove these hypotheses.

## 4 Conclusion

Ethanol steam reforming using an ethanol to H_2_O mixture of 1:7 was investigated over Al‐doped ZnO catalysts synthesized via coprecipitation. Ethanol dehydrogenation to acetaldehyde prevailed at lower temperatures, while acetone formation coupled with CO_2_ formation dominated at higher temperatures. Acetaldehyde was identified as the key intermediate in the formation of acetic acid, acetone, and propylene. The consecutive reaction scheme aligns with previous mechanistic insights, suggesting that acetaldehyde forms surface acetal species, which are dehydrogenated to acetate species. Decarboxylative ketonization of neighboring acetate species yields acetone and CO_2_ as supported by feeding acetaldehyde and acetic acid as reactants.

Al doping of ZnO increased the specific surface area significantly but did not affect ethanol conversion, pointing to pronounced structure sensitivity. Al doping was found to influence the reaction network in two ways: it favors acetaldehyde formation by lowering its rate of conversion to acetic acid, likely due to the decreased nucleophilicity of the OH groups preventing surface acetal formation, and it alters the by‐product distribution by increasing ethyl acetate formation. Both observations are explained by the increased number of Lewis acidic sites introduced by Al doping, which lower the basicity of the hydroxylated ZnO surface. Throughout all reactions, the CO_2_ to acetone ratio exceeded the expected stoichiometric amount of 1:1, resulting from the decomposition of intermediates and coke gasification.

The deepened insight into the consecutive conversion of ethanol in the presence of steam via acetaldehyde, acetic acid, and its decarboxylative ketonization to acetone enables the rational improvement of ZnO‐based catalysts applied in the synthesis of acetaldehyde and acetone.

## 5 Experimental Section

5.1

The hydrozincite precursor was obtained via coprecipitation from a 1 M metal nitrate solution (Zn(NO_3_)_2_ · 6 H_2_O, > 99.0%, and Al(NO_3_)_3_ · 9 H_2_O, 99.997%, both Sigma–Aldrich) using a 1.6 M Na_2_CO_3_ (> 99.5%, Sigma–Aldrich) solution as the precipitating agent [25]. The precipitation was carried out with 110 mL of purified water (HPLC‐grade) prefilled in a double‐walled reactor maintained at 65°C using a thermostat. The reagents were added continuously at a rate of 4.2 mL min^−1^ using a peristaltic pump. During precipitation, the pH was kept constant at 6.5 using a TitroLine6000 auto titrator. After precipitation, the resulting white precipitate was left to age for 10 min without pH control at 65°C under continuous stirring. The precipitate was then filtered and washed over a 4 µm frit using 1000– 1200 mL of purified water until the electrical conductivity of the filtrate dropped below 100 µS cm^−1^. The washed precursor was subsequently dried in an oven at 80°C overnight. To obtain the corresponding oxides, the precursor was calcined in synthetic air (40 mL min^−1^) at 320°C using a heating rate of 2 K min^−1^ for 4 h. The resulting light yellow ZnO and ZnO:Al samples were pressed and ground to a particle size of 250–355 µm. In this work, the catalysts are labeled as ‘Al_*x*_ZnO’, where *x* refers to the molar fraction of Al in the doped sample.

**ICP‐MS** The Zn and Al contents in the catalysts were analyzed by inductively coupled plasma mass spectrometry (ICP‐MS) using a PerkinElmer NexION 350 X calibrated to the desired masses. ≈5–10 mg of the catalysts were dissolved in 300 μL of HNO_3_ (65%, Sigma Aldrich) and then diluted with H_2_O (p.a) to a total volume of 10 mL.

**Thermogravimetry** Prior to thermogravimetric (TG) and N_2_ physisorption measurements, the samples were dried in an inert gas flow at 200°C for 2 h. TG analysis was performed using a thermobalance (Cahn TG‐2131) coupled with a quadrupole mass spectrometer (Pfeiffer Vacuum, ThermoStar). 20 mg of the catalyst were heated from room temperature (RT) to 800°C in He at a rate of 5 K min^−1^. The spent catalyst was measured with the same program but using 20% O_2_/He to investigate coke burn‐off.

**N**_**2**_ **physisorption** 70–100 mg of the 250–300 µm sieve fraction were used in an Autosorb‐1C (Quantachrome) at a constant temperature of ≈77 K subsequent to degassing at 200°C for 2 h.

^**27**^ **Al solid‐state NMR** experiments were recorded on a Bruker Avance NEO 400 NMR spectrometer at a magnetic field of 9.34 T at 104.318 MHz using an Bruker 4 mm MAS NMR probe at rotation frequencies of 12.5 kHz. A molar aqueous solution of AlCl_3_ (aq) was used as the external standard. 60.000–120.000 scans were performed with a spectral width of 125 kHz, a pulse length of 0.6 μs (90° pulse length 3.5 μs) for nonselective excitement of all transitions and a repetition time of 0.1 s.

**Pyridine diffuse reflectance infrared Fourier transformed spectroscopy** Fourier transformed infrared (FTIR) spectrometer (Thermo Fisher Scientific Nicolet iS50) was equipped with a Praying Mantis diffuse reflectance mirror geometry (Harrick) and a liquid N_2_‐cooled MCT‐A detector. The spectrometer was continuously purged with dried air (PG28L, Peak Scientific) to minimize moisture. Spectra were recorded from 400 to 4000 cm^−1^ with a resolution of 4 cm^−1^ and 64 scans. Background spectra were recorded prior to pyridine adsorption at 30°C to 150°C in He. Pyridine was adsorbed at 25°C for 30 min by a saturator maintained at 0°C. After flushing with He for 30 min, a temperature‐programmed desorption profile was recorded from 25°C to 150°C at a rate of 2 K min^−1^.

**X‐ray diffraction** A Bruker D8 Discover diffractometer was used with Cu K*α* radiation (1.5406 Å) in the 2*θ* range from 10° to 80° with a step width of 0.03°. The reference patterns were obtained from the crystallography open database. The crystallite size was determined by applying the Scherrer equation to the reflection at ≈46° 2*θ*, where *D* is the crystallite size, *K* the crystallite‐shape factor (0.89), *λ* is the wavelength of the applied X‐ray radiation, *β* is the full width at half maximum (FWHM) in radians, and *θ* is the diffraction angle.

(12) $$D=\;\frac{K\lambda }{\beta \cos\theta }$$

### 5.2 Kinetic Investigations

**SCHEME 2:**
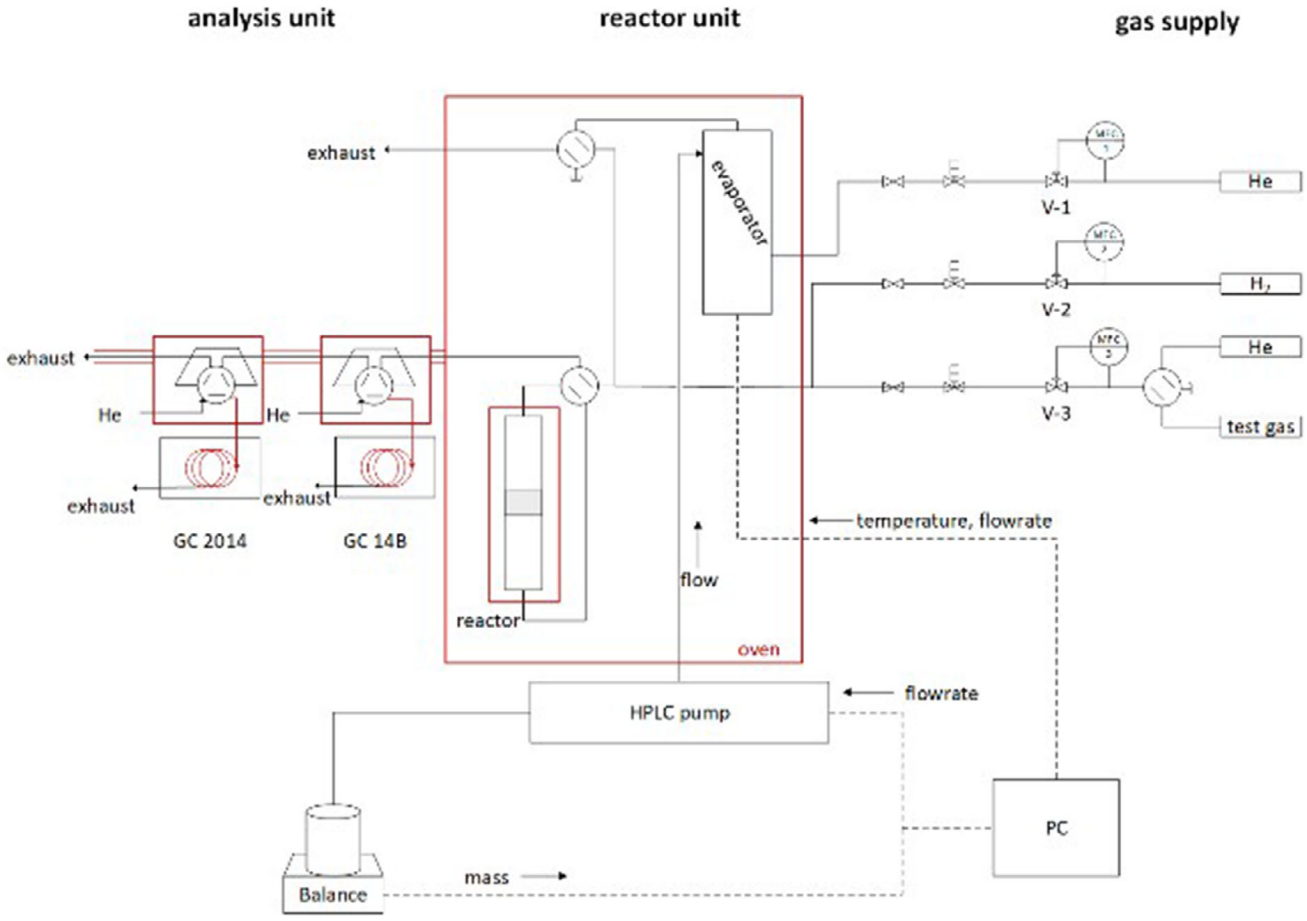
Setup for kinetic ethanol steam reforming measurements.

The setup for the kinetic experiments consisted of an evaporation unit and a quartz glass tube reactor, both placed in an oven set to 120°C (Scheme 2). All tubes were heated to 150°C to prevent condensation. An ethanol‐H_2_O mixture was introduced at the top of the glass evaporator, which was filled with glass beads, using a high‐pressure liquid (HPLC) pump. The evaporator was heated to 120°C with heating cables, and He was introduced at the bottom of the evaporator to transport the vaporized mixture. The quartz glass tube reactor was heated by an electric furnace (HTM Reetz) to a maximum temperature of 600°C, while the catalyst bed temperature was monitored by a thermocouple positioned within the bed. Two online GCs were used to quantify the product gases using a six‐port valve with sample loop. The first GC was equipped with an FID detector and a Restek U‐bond capillary column (30 m, 0.53 mm ID, 20 µm) to quantify small polar molecules. The second GC, equipped with a TCD detector utilized a packed Porapak Q (80/100, 6 ft, 2 mm ID) and a molecular sieve 13X (60/80, 2 m, 2 mm ID) column configuration to quantify permanent gases as well as CO and CO_2_. This dual GC analysis ensured the closure of the carbon balance.

100 mg catalyst with a particle size of 250–355 μm was positioned in the reactor with an inner diameter of 2 mm between two quartz wool plugs. Prior to the actual measurement, the initial ethanol volume fraction was monitored over a period of 2 h with online GC measurements taken every 10 min. For this, an ethanol‐H_2_O (1:7 molar ratio) mixture was fed with a rate of 0.097 mL min^−1^ while the He flow through the evaporator was set to 88 mL min^−1^. The ethanol‐H_2_O feed was then switched offline, and the reactor was switched online to dry the catalyst for 2 h in He (100 mL min^−1^) at 200°C. For the temperature step experiments, the temperature was increased to 350°C and held for 20 min before the ethanol‐H_2_O feed stream was switched online. The temperature was held at 350°C for 3 h, and then raised to 400°C, 420°C, and 450°C, each at a rate of 5 K min^−1^ and held for 3 h. The same procedure was performed using an acetaldehyde‐H_2_O mixture (1:7 molar ratio) and an acetic acid‐H_2_O mixture (1:7 molar ratio). For the long‐term stability test, the catalyst was kept at 400°C for 10 h under continuous reaction conditions. GC measurements were performed every 30 min.

The ethanol conversion *X*_EtOH_ is defined as the volume fraction of ethanol converted *y*_0_ − *y*_*i*_ in relation to the initial ethanol volume fraction y_0_

(13) $$X_{\text{EtOH}}=\frac{y_{0}-y_{i}}{y_{0}}$$

The selectivity *S*_C,p_ to product p is reported as normalized carbon‐based selectivity referring to the number of C‐atoms *N*_C, EtOH_ of converted EtOH

(14) $$S_{C_{i},p}=\frac{y_{p}\cdot N_{C,p}}{\left(y_{0,\text{EtOH}}-y_{\text{EtOH}}\right)\cdot N_{C,\text{EtOH}}}$$

Thus, the maximum achievable selectivity of acetone is 75%.

The WGSR was investigated using a flow setup described by Laudenschleger et al.[56] It consisted of an evaporation unit with a CORI‐FLOW (Bronkhorst), a gas supply, a tube reactor made of stainless steel, and a FTIR is50 (Thermo Fisher) for the analysis of gaseous products. 100 mg of the catalyst was used and dried in N_2_ at 200°C for 2 h. A mixture of CO:H_2_O:N_2_ = 4:4:92 was passed through the reactor at 400°C, 450°C, and 500°C for 60 min each. The reaction rates with respect to CO *r*_CO_ were calculated at 450°C using Equation (15), where *Q* is the total volumetric flow rate, *V*_m_ the molar volume (22.4 l mol^−1^), *y*_0,CO_ the initial CO volume fraction, and *X*_CO_ the CO conversion.

(15) $$r_{\mathrm{CO}}=\frac{Q}{V_{m}}\cdot y_{0,\mathrm{CO}}\cdot X_{\mathrm{CO}}$$

## Supporting Information

Additional supporting information can be found online in the Supporting Information section. **Supporting Fig. S1:** XRD patterns of the hydrozincite precursors. **Supporting Fig. S2:** ^27^Al NMR spectra. **Supporting Fig. S3:** N_2_ physisorption isotherms and derived pore volume distributions. **Supporting Fig. S4:** Pyridine DRIFT spectra at 35°C after pyridine adsorption at 35°C for 30 min and subsequent flushing in He for 30 min. Prior to pyridine adsorption, the catalysts were pretreated in He for 2 h at 200°C. **Supporting Fig. S5:** Volume fractions during the temperature step experiment over pure ZnO. **Supporting Fig. S6:** Conversion and carbon‐based selectivities by feeding EtOH:H_2_O:He =  1:7:92, Q =  100 mL min^‐1^ over different masses of pure ZnO at 400°C. Others include propylene, CH_4_, HOAc, and butenes. **Supporting Fig. S7:** Carbon‐based selectivities and ethanol conversion for various Al‐doped ZnO samples at 400, 420, and 450°C. **Supporting Fig. S8:** CO_2_ areas normalized by the catalyst weight obtained by integration of the MS ion currents at m/z =  44 during TG in 20% O_2_/He using the spent catalysts. **Supporting Fig. S9:** WGSR reaction over pure ZnO using CO:H_2_O =  1:1 mol, Q =  100 ml min‐1. **Supporting Table S1:** Mole fractions in % obtained for ICP‐MS analysis of the precursor catalysts. **Supporting Table S2:** Conversion in % and carbon‐based selectivities in % for all catalysts at 400°C. **Supporting Table S3:** Conversion in % and carbon‐based selectivities in % for all catalysts at 420°C. **Supporting Table S4:** Conversion in % and carbon‐based selectivities in % for all catalysts at 450°C. **Supporting Table S5:** Acetaldehyde conversion in % and carbon‐based selectivities in % for feeding acetaldehyde. **Supporting Table S6:** Acetic acid conversion in % and carbon‐based selectivities in % for feeding acetic acid.

## Funding

This study was supported by BMFTR (03XP0599B).

## Conflicts of Interest

The authors declare no conflicts of interest.

## Supporting information

Supplementary Material

## Data Availability

The data that support the findings of this study are available from the corresponding author upon reasonable request.
