# Supplementary material for: Steam Reforming of Ethanol to Acetaldehyde and Acetone Over Al‐Doped ZnO Catalysts
Source: ChemSusChem. 2026 Jan 5;19(1):e202501925. doi: 10.1002/cssc.202501925 (PMC12766874; doi:10.1002/cssc.202501925)
Supplement: Supplementary file 1 — Supplementary Material [file CSSC-19-e202501925-s001.pdf]

WILEY-VCH

Supporting information

Steam reforming of ethanol to acetaldehyde and acetone over Al-doped ZnO catalysts

Astrid Sophie Müller[a], Lars Malte Alfes[a], Michael Fechtelkord[b], and Martin Muhler[a]*

[a] A. S. Müller, L. M. Alfes, Prof. Dr. M. Muhler
Laboratory of Industrial Chemistry
Department of Chemistry and Biochemistry
Ruhr University Bochum
D-44780 Bochum
E-mail: martin.muhler@ruhr-uni-bochum.de

[b] Prof. Dr. M. Fechtelkord
Geoscience solid state NMR spectroscopy
Ruhr University Bochum
D-44780 Bochum

WILEY-VCH

Table S1. Mole fractions in % obtained for ICP-MS analysis of the precursor catalysts.

Catalyst	x(Zn) / %	x(Al) / %
ZnO	100.0	0
$Al_{0.02}ZnO$	97.8	2.2
$Al_{0.03}ZnO$	96.9	3.1
$Al_{0.04}ZnO$	95.8	4.2
$Al_{0.05}ZnO$	94.6	5.4
$Al_{0.06}ZnO$	93.1	6.9
$Al_{0.10}ZnO$	89.5	10.5

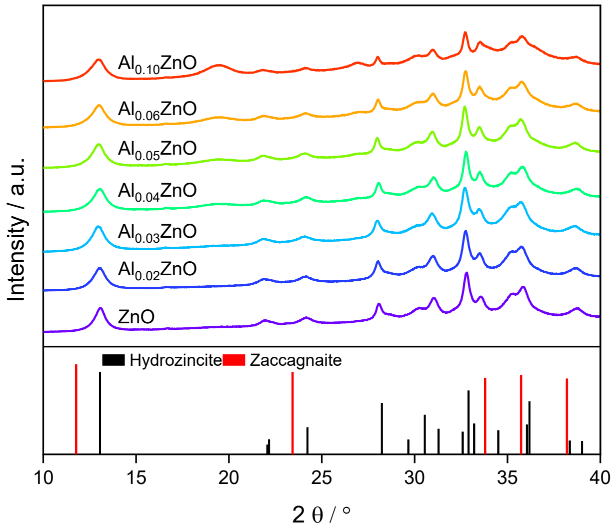

Figure S1. XRD patterns of the hydrozincite precursors.

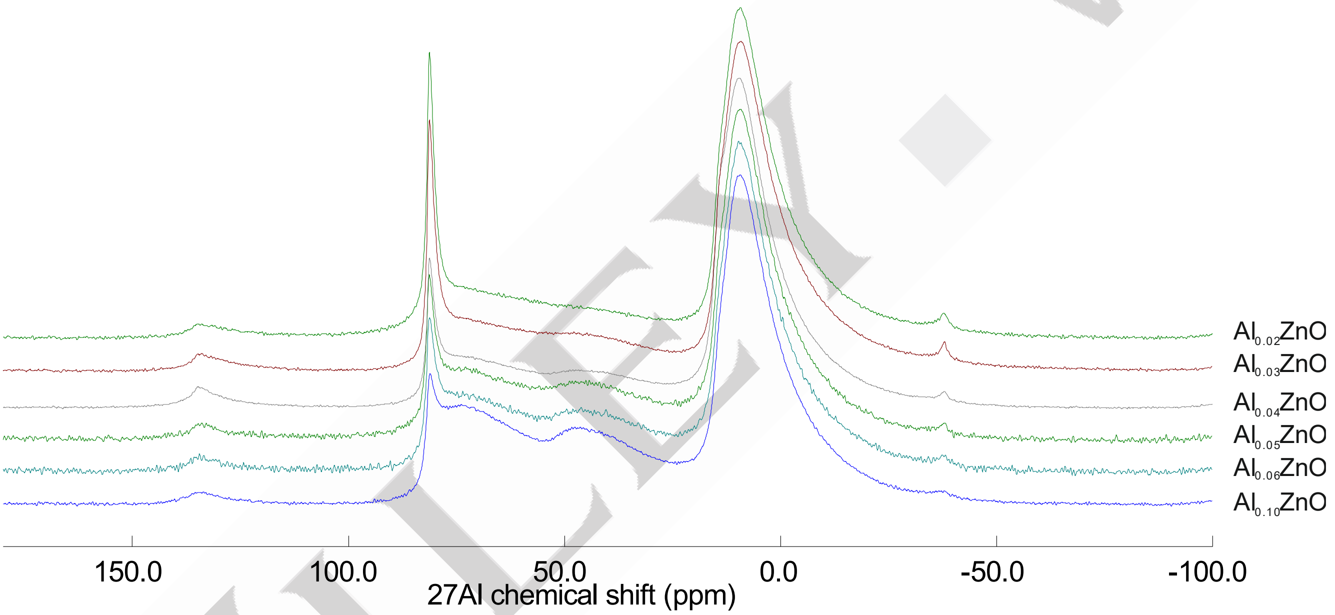

Figure S2. ^{27}Al NMR spectra.

WILEY - VCH

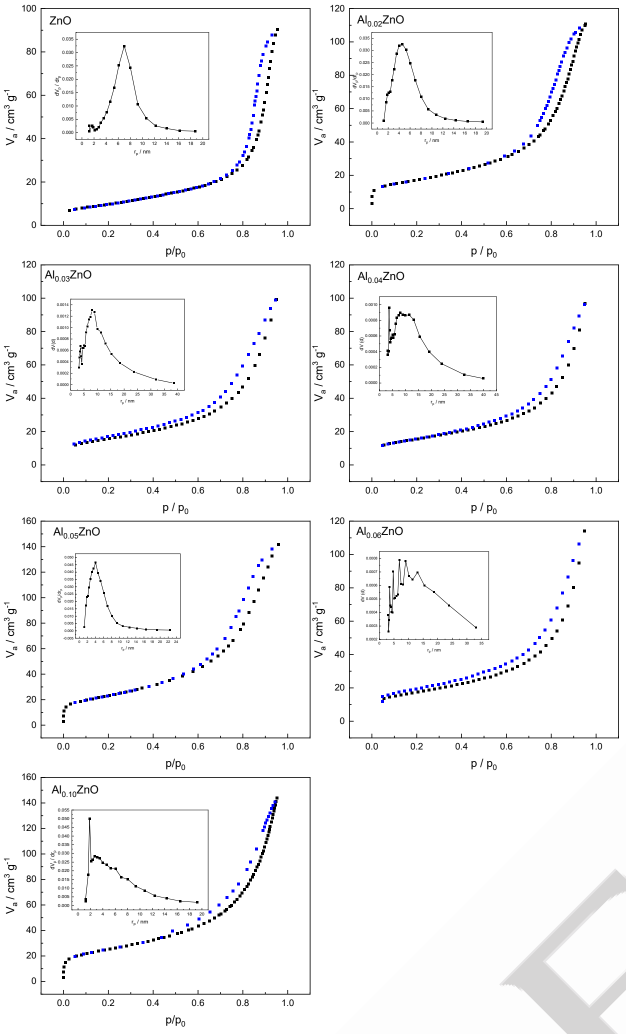

Figure S3. N_2 physisorption isotherms and derived pore volume distributions.

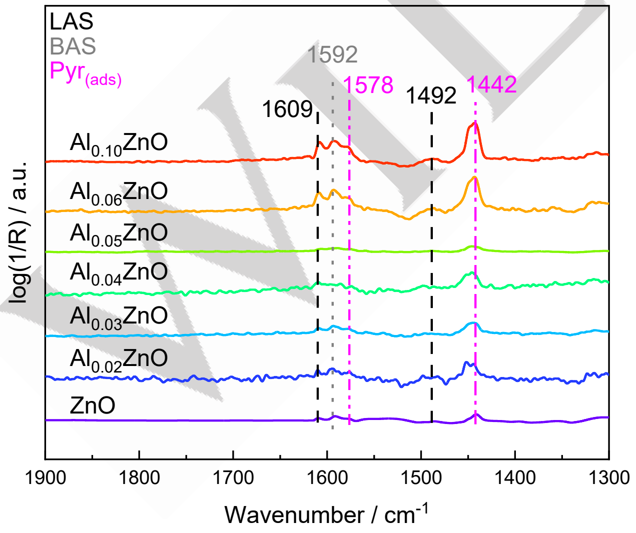

Figure S4. Pyridine DRIFT spectra at 35 °C after pyridine adsorption at 35 °C for 30 min and subsequent flushing in He for 30 min. Prior to pyridine adsorption, the catalysts were pretreated in He for 2 h at 200 °C.

WILEY-VCH

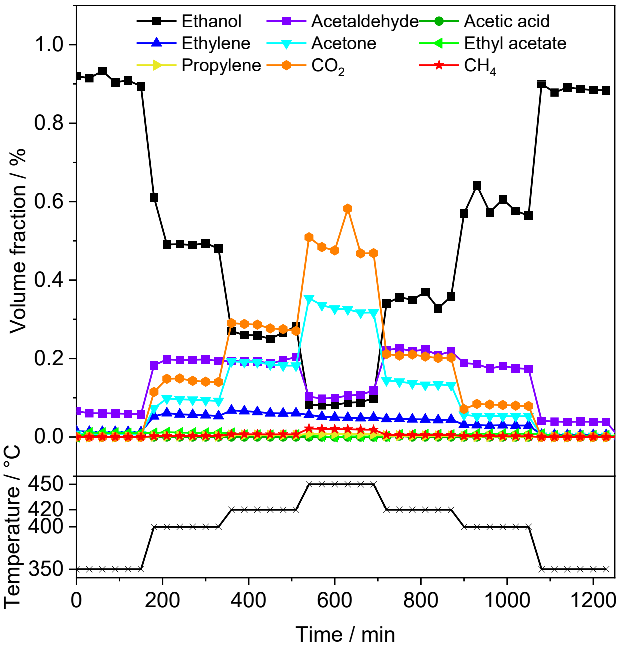

Figure S5. Volume fractions during the temperature step experiment over pure ZnO.

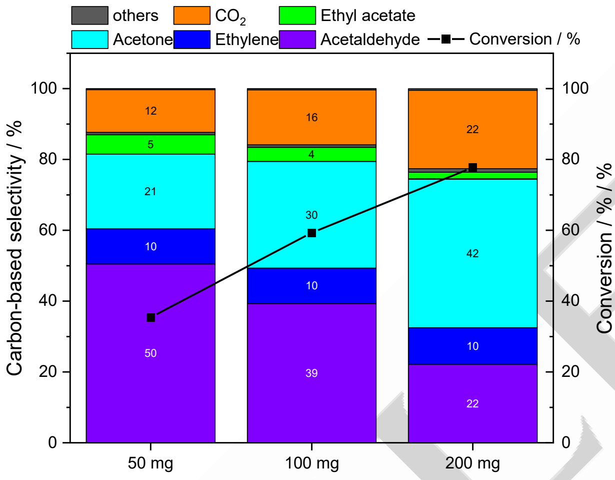

Figure S6. Conversion and carbon-based selectivities by feeding EtOH:H_2O:He = 1:7:92, Q = 100 mL min^{-1} over different masses of pure ZnO at 400 °C. Others include propylene, CH_4, HOAc, and butenes.

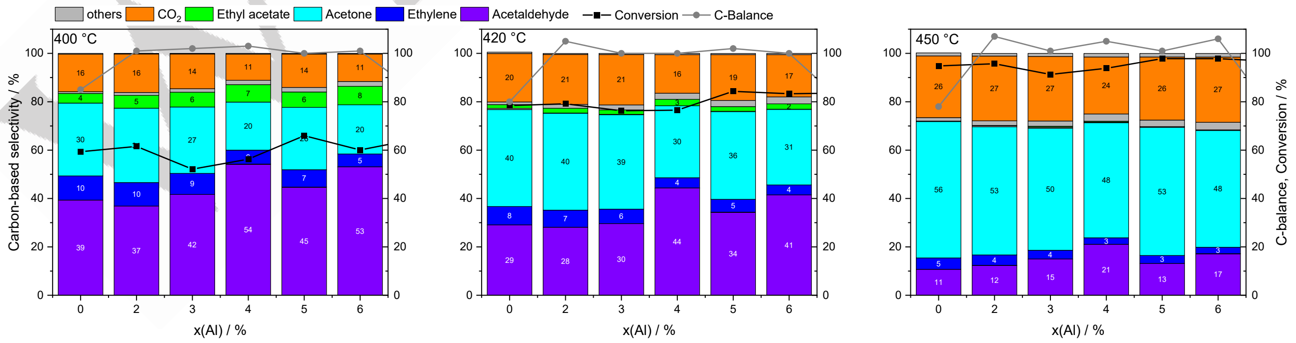

Figure S7. Carbon-based selectivities and ethanol conversion for various Al-doped ZnO samples at 400, 420, and 450 °C.

WILEY · VCH

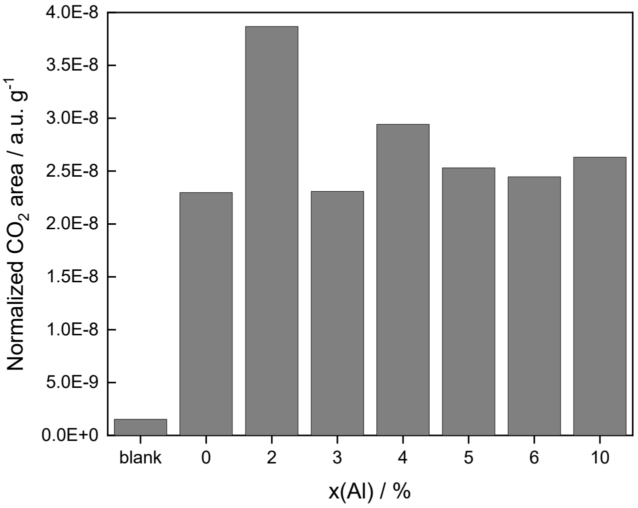

Figure S8. CO_2 areas normalized by the catalyst weight obtained by integration of the MS ion currents at m/z = 44 during TG in 20 % O_2/He using the spent catalysts.

TG measurements were performed using the spent catalysts in 20% O_2/He. The ion currents at m/z = 44 were integrated during the heating to 800 °C. Figure S8 shows the integrated areas of mass 44 normalized to the weight of the samples. The resulting value is proportional to the amount of CO_2 released during heating in O_2.

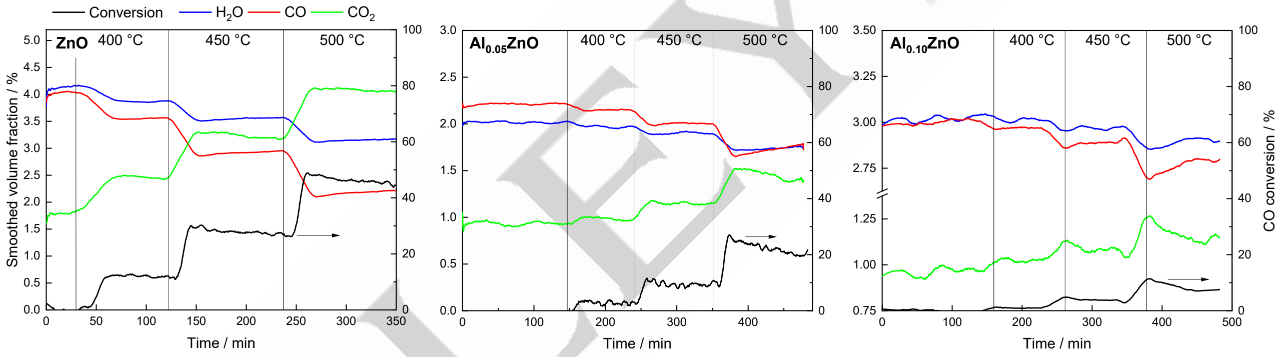

Figure S9. WGSR reaction over pure ZnO using CO:H_2O = 1:1 mol, Q = 100 ml min^{-1}.

Tables S2, S3, and S4 contain the conversion and carbon-based selectivites for all catalysts used. HOAc refers to acetic acid. Since it was not possible to distinguish between different C_4 olefines using the FID GC, they were summarized in “Butenes” (e.g. 1-butene, isobutene).

Table S2. Conversion in % and carbon-based selectivities in % for all catalysts at 400 °C.

Catalyst	X	C_2H_4O	C_3H_6O	CO_2	C_2H_4	C_3H_6	$C_4H_8O_2$	CH_4	HOAc	Butenes
ZnO	59.3	39.3	30.1	15.5	10.0	0	4.0	0.3	0	0
$Al_{0.02}ZnO$	61.5	36.9	30.8	15.9	9.7	1.2	5.3	0.2	0	0
$Al_{0.03}ZnO$	52.1	41.7	27.5	14.3	8.7	1.5	6.1	0.3	0	0
$Al_{0.04}ZnO$	56.3	54.2	20.0	10.8	5.7	1.8	7.2	0.3	0.09	0
$Al_{0.05}ZnO$	66.0	44.7	26.0	13.8	7.2	1.9	6.4	0.3	0.03	0
$Al_{0.06}ZnO$	60.1	53.1	20.3	11.3	5.3	2.0	7.7	0.3	0.06	0
$Al_{0.10}ZnO$	64.6	63.8	13.8	8.1	3.7	2.2	8.1	0.3	0	0

Table S3. Conversion in % and carbon-based selectivities in % for all catalysts at 420 °C.

Catalyst	X	C_2H_4O	C_3H_6O	CO_2	C_2H_4	C_3H_6	$C_4H_8O_2$	CH_4	HOAc	Butenes
ZnO	78.4	29.0	40.0	20.0	7.5	1.1	1.5	0.5	0	0
$Al_{0.02}ZnO$	79.2	28.1	40.0	20.7	7.1	1.6	2.0	0.4	0	0.1
$Al_{0.03}ZnO$	76.2	29.6	39.0	20.8	6.0	2.1	1.8	0.5	0	0.1
$Al_{0.04}ZnO$	76.5	44.3	29.8	16.0	4.2	2.5	2.6	0.5	0.02	0
$Al_{0.05}ZnO$	84.3	34.3	36.3	19.0	5.3	2.6	2.0	0.5	0.01	0.1
$Al_{0.06}ZnO$	83.3	41.5	31.2	17.5	4.1	2.9	2.2	0.5	0.04	0.1
$Al_{0.10}ZnO$	83.6	54.8	22.3	13.1	2.9	3.1	3.2	0.6	0.10	0

WILEY-VCH

Table S4. Conversion in % and carbon-based selectivities in % for all catalysts at 450 °C.

Catalyst	X	C_2H_4O	C_3H_6O	CO_2	C_2H_4	C_3H_6	$C_4H_8O_2$	CH_4	HOAc	Butenes
ZnO	94.8	10.7	56.2	25.4	4.7	1.4	0	1.2	0	0.3
$Al_{0.02}ZnO$	95.7	12.3	53.0	26.7	4.3	1.9	0.2	1.1	0.09	0.5
$Al_{0.03}ZnO$	91.3	15.0	50.5	26.7	3.6	2.2	0.4	1.3	0	0.4
$Al_{0.04}ZnO$	93.8	21.1	47.6	23.6	2.7	3.0	0.4	1.5	0	0.2
$Al_{0.05}ZnO$	97.8	13.2	53.0	26.1	3.3	2.7	0	1.4	0	0.3
$Al_{0.06}ZnO$	97.8	17.1	48.3	27.0	2.6	3.1	0	1.5	0.07	0.3
$Al_{0.10}ZnO$	96.7	30.8	39.3	22.2	2.1	3.8	0	1.6	0	0.4

Table S5. Acetaldehyde conversion in % and carbon-based selectivities in % for feeding acetaldehyde.

Temperature	X	C_3H_6O	CO_2	HOAc	C_3H_6	C_2H_4	Butenes	EtOH
350 °C	14.2	58.7	33.2	5.7	1.1	0.0	0.0	1.2
400 °C	48.5	62.5	33.4	1.5	1.7	0.5	0.0	0.4
420 °C	63.3	63.4	32.6	1.2	1.8	0.7	0.2	0.1
450 °C	81.6	64.0	32.8	0.1	1.5	1.2	0.4	0

WILEY-VCH

Table S6. Acetic acid conversion in % and carbon-based selectivities in % for feeding acetic acid.

Temperature	X	C_3H_6O	CO_2	Butenes	CH_4
350 °C	79.0	73.0	27.0	0	0
400 °C	95.3	73.8	25.9	0.02	0.24
420 °C	98.4	75.5	23.9	0.20	0.47
450 °C	>99.9	71.0	27.2	0.65	1.15

WILEY-VCH
